# Acute postoperative pain control in pediatric patients: a scoping review

**DOI:** 10.12701/jyms.2026.43.1

**Published:** 2025-12-10

**Authors:** Eun Kyung Choi, Sang-Jin Park, Seong Wook Hong

**Affiliations:** 1 Department of Anesthesiology and Pain Medicine, Yeungnam University College of Medicine, Daegu, Korea; 2 Department of Anesthesiology and Pain Medicine, School of Medicine, Kyungpook National University, Daegu, Korea

**Keywords:** Analgesics, Pain, Postoperative, Pediatrics, Pain measurement

## Abstract

Acute postoperative pain results from tissue injury during surgery and subsequent inflammatory responses. The incidence of chronic postsurgical pain ranges from 10% to 30%, and its development is influenced by various clinical factors, including psychological, biological, and social determinants. Optimal management of acute postoperative pain is crucial for enhancing patient satisfaction, preventing adverse outcomes in the immediate postoperative period, and minimizing progression to chronic postoperative pain. In particular, postoperative pain in pediatric patients is often underestimated and inadequately managed because of developmental differences in pain perception, expression, and challenges in assessment. Therefore, age-appropriate and validated assessment tools that consider cognitive development and situational factors are required. Given age-related variability in pharmacokinetics and pharmacodynamics, individualized multimodal analgesic strategies with careful dose adjustments should be utilized. These approaches have demonstrated improved analgesic efficacy and enhanced recovery outcomes in pediatric surgical patients. A comprehensive understanding of pediatric pain pathophysiology, combined with appropriate methods of pain assessment and management strategies, should be selected to promote postoperative recovery and reduce morbidity.

## Introduction

Acute postoperative pain is a common consequence of surgical procedures, primarily resulting from tissue injury and subsequent inflammatory responses, and typically persists throughout the immediate recovery period. However, the incidence of chronic postsurgical pain ranges from 10% to 30% and its development is influenced by various clinical factors [1]. The factors contributing to the transition from acute to chronic pain are psychological, biological, and social; additional factors include the type of surgical procedure, severity of preoperative pain, and strategies employed for perioperative pain management [2-4]. Therefore, effective acute postoperative pain management is crucial for enhancing patient satisfaction, preventing adverse outcomes in the immediate postoperative period, and minimizing progression to chronic postoperative pain.

Although postoperative pain management is well-established in adult patients, it is frequently underestimated or undertreated in pediatric populations [5]. Pediatric patients frequently experience difficulty verbally expressing their pain; consequently, specialized scale assessment tools should be used to evaluate postoperative pain. Therefore, based on a comprehensive understanding of the pathophysiology of pain in pediatric patients, appropriate methods for pain assessment and management strategies should be selected to promote postoperative recovery and reduce morbidity.

## Pathophysiology of postoperative pain

Pain is a complex process involving multiple components that are activated by mechanical, chemical, and thermal stimuli. These nociceptive stimuli can elicit inflammatory responses with various mediators such as prostaglandins, bradykinin, and cytokines, which cause somatic, visceral, or neuropathic pain. Peripheral sensitization by noxious stimuli leads to primary hyperalgesia at the injury site [6], and central sensitization induced by sustained nociceptive signaling can exacerbate clinical manifestations characterized by secondary hyperalgesia [6] or chronic neuropathic pain [7]. Inadequate management of acute pain in children can worsen pain perception because of immaturity of the descending inhibitory pathways and developmental characteristics of neuroplasticity [8]. All components except the sensory component, namely the physiological, behavioral, and cognitive components, are also associated with pain experience. Genetic, developmental, and environmental factors further influence pain perception and analgesia [9]. Therefore, an appropriate assessment of postoperative pain and the application of individualized multimodal analgesic approaches in pediatric patients ensure effective pain management and improve recovery outcomes.

Multimodal analgesia has been implemented in pediatric patients to address acute pain in the perioperative period and to optimize analgesic efficacy while minimizing opioid-related adverse effects. In this approach, pharmacologic agents (i.e., basic analgesia, opioids, and adjuvant analgesia) with different mechanisms of action are combined with nonpharmacological modalities, including regional anesthesia, psychological interventions, distraction techniques, and rehabilitation [10]. Unlike adults, children exhibit age-dependent variations in drug metabolism, pain perception, and communication; therefore, children require age-specific strategies and careful dose adjustments [11,12]. Thus, multimodal analgesia improves postoperative pain control in pediatric populations, facilitates faster recovery, and enhances overall patient and caregiver satisfaction [13,14].

## Acute pain assessment in pediatric patients

An adequate pediatric pain assessment tool (Table 1) that considers age, cognitive level, and pain-inducing situations should be selected because pain expression in children is often unclear and its evaluation remains difficult. Accordingly, the following option-based evaluation methods are commonly employed: (1) biological measures that rely on physiological parameters (e.g., heart rate [HR], blood pressure [BP], and respiratory rate); (2) observational measures based on a child’s reaction to pain (e.g., motor response, facial expression, and crying); and (3) self-report measures based on the description of pain perception. Behavioral assessment methods serve as valuable approaches for evaluating nonverbal children. For instance, the Children’s Hospital of Eastern Ontario Pain Scale is widely used to assess pain in children [3]. Several other approaches, such as the CRIES and Neonatal Infant Pain Scale (NIPS), have been utilized to assess pain in neonates and infants [15,16]. The CRIES scale is used to evaluate the following five items: crying, increasing oxygen requirement, increased vital signs (HR/BP), expression (facial), and sleeplessness [15]. The NIPS consists of six parameters: facial expression, crying, breathing pattern, arms, legs, and state of arousal [16]. The Premature Infant Pain Profile is specifically used to assess pain in preterm infants, in which four items are evaluated: gestational age; behavioral state; HR and oxygen saturation changes; and brow bulge, eye squeeze, and nasolabial furrow [17]. The Face, Legs, Activity, Cry, and Consolability (FLACC) scale is utilized to examine the intensity of pain or discomfort after surgery or procedures in children aged <3 years [18]. When children reach an age at which they can describe pain characteristics (3–7 years), pain can be assessed using observational scales as well as self-report scales, such as the Objective Pain Scale and the Comfort Scale [19]. Self-report measures are most appropriate for older pediatric patients who can express a subjective sensation of pain [20]. Using self-report measures, such as direct questioning, pictorial methods, and self-rating scales, requires appropriate cognitive and linguistic development. The Visual Analog Scale is a commonly used method through which patients subjectively rate their pain from 0 (no pain) to 10 (most severe pain). Complementing this approach, the Wong-Baker FACES Pain Rating Scale is a representative pictorial scale that presents visual materials such as facial drawings, colors, or shapes and allows children to select the image that best represents their pain [21].

## Preoperative considerations

The management of acute postoperative pain begins preoperatively, based on the anticipated intensity and duration of postoperative pain. In pediatric patients, the most frequently performed surgical procedures are often accompanied by substantial pain, although these procedures are minimally invasive. Procedures such as tonsillectomy, appendectomy, orchidopexy, and various other orthopedic surgeries cause greater pain than anticipated [22]. Therefore, children and their parents should be provided with clear explanations of the surgical procedure and available treatment options, including pharmacological and nonpharmacological approaches.

Preemptive analgesia refers to antinociceptive strategies that involve the administration of analgesic interventions before the onset of a noxious stimulus [23]. The primary goal is to inhibit central sensitization, thereby reducing the intensity of postoperative pain and minimizing the need for additional rescue analgesia [23]. Preemptive approaches include pharmacological methods, such as opioids, nonsteroidal anti-inflammatory drugs (NSAIDs), acetaminophen, local anesthetics, and *N*-methyl-D-aspartate (NMDA) receptor antagonists, as well as nonpharmacological techniques, including regional nerve blocks, local anesthesia, cryotherapy or thermotherapy, and psychological preparation [24].

## Preoperative pharmacological interventions

Analgesics are the primary agents used in pain management. The selection and administration of analgesics according to pain should be individualized, considering the pain relief and potential side effects. The World Health Organization has presented a flexible approach to analgesic choice based on pain intensity through the concept of an analgesic ladder [18]. This suggests that the combination of opioids, nonopioids, and adjuvant analgesics depends on whether the pain is mild, moderate, or severe. Pediatric patients often experience anxiety, fear of separation, and stress related to unfamiliar environments more intensely than adults do. Preoperative anxiety can negatively influence perioperative cooperation, delay recovery, increase postoperative pain, and contribute to adverse hospital-related memories in the long term [25]. Therefore, premedication can considerably alleviate anxiety, facilitate smooth induction of anesthesia, and promote postoperative recovery in children. Midazolam is the most common premedication for pediatric anesthesia because of its rapid onset, anxiolytic properties, and ability to facilitate smoother anesthetic induction. Various studies have demonstrated that it effectively reduces preoperative anxiety and improves perioperative adherence [26,27]. However, increased doses have been associated with adverse events including respiratory depression and prolonged postoperative sedation; therefore, careful dose titration and monitoring are necessary.

## Preoperative nonpharmacological interventions

Nonpharmacological preoperative interventions include distraction-based interventions and relaxation techniques such as music, games, movies, video games, and virtual reality [28,29]. They serve as alternatives to pharmacological interventions to alleviate anxiety, improve cooperation of children, and relieve perioperative pain [30]. The underlying mechanism is thought to involve cognitive and sensory distraction, which reduces the child’s focus on anxiety-provoking stimuli and pain sensations, thereby promoting a more positive perioperative experience. Recent evidence has supported the efficacy of these approaches. Jung et al. [31] demonstrated that virtual reality distraction is an effective nonpharmacological approach for mitigating perioperative anxiety in pediatric patients undergoing surgery. Similarly, Sunitha Suresh et al. [32] provided empirical support for audio therapy modalities, such as music and audiobooks, in decreasing postoperative pain intensity in pediatric patients following major surgeries. Their findings suggested that these auditory interventions can serve as effective, low-risk adjuncts to conventional analgesic regimens, potentially reducing the reliance on pharmacological pain management and its associated side effects. Additionally, the presence of parents during induction has been proposed as a strategy to reduce patient anxiety. Arai et al. [33] demonstrated that parental presence during anesthesia induction potentiates the efficacy of preoperative midazolam in mitigating the emergence of behavioral disturbances in pediatric patients. In addition, parental presence, video distraction, and a combination of both have comparable effects in reducing preoperative anxiety and postoperative behavioral disturbances in pediatric patients [34].

## Intraoperative consideration

### 1. Hypnotic agents for general anesthesia

Inhalational anesthetics (such as sevoflurane and desflurane) and intravenous anesthetics (such as propofol) are commonly used as hypnotic agents for general anesthesia in pediatric patients. They do not act directly on the nociceptive pathways; instead, they decrease pain perception by causing patients to become unconscious. Numerous studies have investigated the relationship between hypnotic agents and postoperative pain in pediatric patients [35,36]. Abdallah et al. [35] conducted a meta-analysis of 13 randomized controlled trials and showed that children who received sevoflurane for anesthesia maintenance had a higher incidence of postoperative pain than those who received propofol. Additionally, the frequency of rescue analgesic use was higher in the sevoflurane group than in the propofol group. Although inhalational agents such as sevoflurane and desflurane are widely used because of their rapid onset and recovery profiles, they may also contribute to postoperative hyperalgesia [36]. Two mechanisms have been proposed to explain this phenomenon: (1) modulation of NMDA receptor activity and altered nociceptive processing, which transiently enhances pain sensitivity after anesthesia, and (2) enhancement of excitatory neurotransmitters in the spinal cord to possibly promote central sensitization and amplify nociceptive signaling. Therefore, the anesthetic choice and proactive postoperative pain management strategies should be carefully considered in pediatric patients receiving general anesthesia for surgical interventions.

### 2. Antinociceptive agents

#### 1) Opioids

Opioids are primary antinociceptive agents used for the management of surgical pain. They target multiple classes of receptors in the brain, spinal cord, and peripheral tissues [37,38]. These interactions with opioid receptors disrupt the transmission of nociceptive circuits through two main mechanisms: (1) blockade of peripheral pain transmission to the spinal cord and (2) enhancement of the descending inhibition of nociception, beginning at the periaqueductal gray [9-16]. Although these two mechanisms decrease pain information processing, opioid activity within the amygdala attenuates nociceptive perception and the associated affective responses to pain [39,40]. Opioids also increase cholinergic input to the sinoatrial node, consequently inducing bradycardia and attenuating sympathetic responses to nociception [41]. Opioids are classified as agonists, partial agonists, or mixed agonist-antagonists based on their interaction with receptor subunits. The agonists commonly used in children include morphine, hydromorphone, and fentanyl (Table 2) [18,42]. Morphine, a prototypical opioid agonist of moderate potency, has a half-life of approximately 2 hours and a pharmacological activity duration of approximately 5 hours. Because it is metabolized in the kidney, its duration of action may be prolonged in patients with renal impairment [43]. Hydromorphone, a semisynthetic opioid, has a more rapid onset of action and is four to six times more potent than morphine. It can be administered to patients with renal impairment and is particularly useful in cases of opioid tolerance [43]. Fentanyl, a synthetic opioid, is approximately 50 to 70 times more potent than morphine; however, its duration of action is relatively short, lasting approximately 1 hour. Transdermal formulations may serve as an alternative for pain management, and intravenous administration provides a particularly rapid onset of analgesia, which can be effectively used to manage acute postoperative pain in the post-anesthesia care unit (PACU) [44]. Opioid agonists lack a ceiling effect, and dosing should be guided by a balance between increasing analgesia and dose-related adverse effects. Common adverse effects include physical dependence, nausea, vomiting, respiratory depression, oversedation, pruritus, constipation, and urinary retention [45]. Opioids should be carefully prescribed to pediatric and adolescent populations because of concerns regarding adverse effects, potential misuse, and accidental ingestion. Considering these adverse effects and precautions, opioids should be used from a multimodal perspective, recognizing the pharmacological properties of analgesic agents.

#### 2) Nonopioids

Mild-to-moderate postoperative pain is commonly managed with nonopioid drugs, such as NSAIDs and acetaminophen. These analgesics are also used as part of a multimodal analgesic strategy for managing acute pain in pediatric patients [46,47]. NSAIDs reduce pain and inflammation by inhibiting cyclooxygenase (COX). When administered in combination with opioids, they offer the advantage of an opioid-sparing effect [48]. However, NSAIDs are associated with adverse effects such as gastropathy, renal impairment, and increased bleeding tendency [48]. The representative NSAID commonly used in pediatric patients is ibuprofen, the most common NSAID for acute pain management; it is the only NSAID approved for use in children aged ≥6 months [49]. It can also reduce opioid requirements [50]. In terms of safety and tolerability, it is associated with fewer adverse effects (e.g., nausea, vomiting, and dizziness) than opioid analgesics [51]. Acetaminophen is another widely used analgesic in the pediatric population. It weakly inhibits COX, resulting in negligible anti-inflammatory activity, but elicits analgesic and antipyretic effects [52]. Acetaminophen is often preferred over NSAIDs because of its relatively low incidence of adverse effects; however, it should be administered cautiously to pediatric patients with renal or hepatic impairment [53]. It is available in multiple formulations, including oral, rectal, and intravenous. During the perioperative period, parenteral administration is generally preferred owing to its rapid onset, reliable bioavailability, and suitability for patients who are nil per os or have compromised gastrointestinal function [54]. Alhashemi and Daghistani [55] showed that acetaminophen reduced postoperative opioid consumption in pediatric patients. Furthermore, Lee et al. [56] demonstrated that the concomitant administration of acetaminophen and ibuprofen decreased postoperative analgesic consumption compared to either agent alone. Commonly used nonopioid analgesics are summarized in Table 3 [57-59].

#### 3) Ketamine

Ketamine, a phencyclidine derivative, causes sedation, amnesia, and analgesia. It has been widely used in pediatric populations. Its antinociceptive effect is attributed to the blockade of NMDA glutamate receptors located on peripheral afferent nociceptive neurons. Consequently, the entry of pain signals into the spinal cord is impeded [60]. Ketamine also contributes to analgesia by blocking NMDA receptors in the cerebral cortex and other arousal system regions [61]. Meta-analyses of the effects of ketamine on postoperative pain in pediatric patients have shown that perioperative administration of ketamine reduces pain intensity in the PACU and decreases the requirement for rescue analgesics [62]. Despite its favorable safety profile, the use of ketamine has been associated with the potential for postoperative emergence delirium, hallucinations, hypertension, tachycardia, dose-dependent respiratory depression, and nausea.

#### 4) Dexmedetomidine

Dexmedetomidine, an α2-adrenoceptor agonist, has sedative, analgesic, and anxiolytic effects. Importantly, it causes minimal respiratory depressant effects and several studies have indicated a reduction in postoperative analgesic requirements [63]. However, its use may be related to adverse events such as bradycardia and hypotension. Dexmedetomidine exerts its analgesic effects via two pathways. In the first, inhibitory neurons are activated in the dorsal horn of the spinal cord by enhancing the descending inhibition of nociceptive transmission [64]. The second pathway involves arousal reduction, whereby analgesia is achieved by suppressing the release of norepinephrine from locus coeruleus neurons to the thalamus and cortex [61]. Previous studies on postoperative pain management demonstrated that the perioperative use of dexmedetomidine effectively reduced postoperative pain and analgesic requirements [65,66]. Lopez et al. [65] showed that dexmedetomidine provided superior postoperative pain control in children undergoing dental bone graft surgery. Chiang et al. [66] performed a meta-analysis and revealed that the use of dexmedetomidine in pediatric patients undergoing strabismus surgery effectively reduced not only postoperative pain but also emergence agitation, postoperative nausea, and vomiting.

### 3. Neuraxial techniques

Neuraxial blocks offer several advantages in the perioperative setting. These techniques provide effective and reliable surgical or postoperative analgesia and attenuate the neuroendocrine stress response to surgery [44]. However, the anatomical and physiological properties of the spinal cord and its enveloping structures in children differ from those in adults [67,68]. The epidural space contains less fat and a thinner ligamentum flavum and dura mater in children than in adults; consequently, the risk of inadvertent dural puncture in children increases. The volume of cerebrospinal fluid is considerably larger in infants and young children than in adults, leading to a wider distribution of intrathecal drugs. In addition, the spinal cord and dural sac terminate at L1 to L2 and S1 to S2 levels in adults, respectively; conversely, they terminate at approximately L3 and S3 to S4 levels in infants. Therefore, a lower interspace approach, such as at L4 to L5 or L5 to S1, is recommended to minimize the risk of spinal cord injury during neuraxial procedures in infants [68]. Although neuraxial blocks are generally safe in pediatric patients, potential complications including neurological injury, local anesthetic systemic toxicity, inadvertent dural puncture, and infection may occur [44].

In the context of postoperative pain control, intrathecal administration of local anesthetics or opioids elicits prolonged analgesic effects [47]. Ganesh et al. [69] demonstrated that the intrathecal administration of morphine can produce sustained postoperative analgesia for up to 24 hours. Similarly, Gall et al. [70] showed that the use of intrathecal morphine was associated with reduced total postoperative opioid consumption via patient-controlled analgesia (PCA). Moreover, the intrathecal administration of nonopioid adjuvants in pediatric patients can contribute to effective postoperative pain relief [71]. Fares et al. [71] showed that the postoperative FLACC pain scores of pediatric patients who underwent abdominal surgery and received intrathecal bupivacaine combined with dexmedetomidine were significantly lower than those of patients who received bupivacaine alone, and patients who received combination treatment had a prolonged time to their first analgesic request.

Epidural anesthesia or analgesia can result in an enhanced titration of analgesia and prolonged pain control during the postoperative period. Continuous epidural infusion of local anesthetics, opioids, and other adjuvants can enhance the efficacy of analgesia and reduce side effects [67]. In pediatric patients undergoing spinal surgery, epidural analgesia using a combination of bupivacaine and hydromorphone provides superior postoperative pain control compared to intravenous PCA with hydromorphone alone [72]. A retrospective study including 63 infants found that thoracic epidural analgesia reduced postoperative opioid use, intensive care unit admissions, and oxygen requirements compared with systemic opioids [73].

### 4. Peripheral nerve blocks

Perioperative peripheral nerve blocks are increasingly used in children. The clinical applicability of peripheral nerve blocks has been expanded through ultrasound application by improving the accuracy of needle placement, reducing the incidence of block failure, and minimizing the risk of systemic local anesthetic toxicity with reduced anesthetic volumes [74]. Commonly used local anesthetics for pediatric peripheral nerve blocks are shown in Table 4 [75]. Nerve blocks have several advantages, including effective postoperative analgesia, reduced opioid consumption, accelerated recovery of gastrointestinal motility, and attenuated surgical stress response [76]. The safety and efficacy of peripheral nerve blocks have also been demonstrated in pediatric patients [77-79]. However, they cause various complications, the most frequently reported of which are block failure, catheter-related issues such as dislodgement and occlusion, and minor local infections. Vascular punctures and transient neurological symptoms are less commonly observed, whereas permanent nerve injury remains exceedingly rare [80,81]. In pediatric patients, peripheral nerve blocks are often performed under general anesthesia or deep sedation to facilitate patient cooperation and allow for greater precision during block placement. Moreover, the pharmacodynamic effects of local anesthetics tend to be more pronounced because of the smaller caliber of peripheral nerves in children [82,83]. Peripheral nerve blocks can be administered as single injections or continuous infusions via a catheter. Dadure et al. [78] demonstrated that continuous peripheral nerve blocks with ropivacaine or bupivacaine improved the analgesic efficacy in pediatric patients undergoing orthopedic surgery. The most common minor complications were catheter-related mechanical problems.

## Postoperative consideration

### 1. Patient-controlled analgesia

PCA refers to the temporary administration of analgesics according to the patient’s individualized demand. In most cases, a continuous basal infusion is supplemented by demand-triggered bolus doses [43]. Generally, PCA can effectively manage pain in children >6 years old [84]. In younger patients or those unable to comprehend the concept, nurse- or parent-controlled analgesia may be more appropriate [18]. PCA is advantageous because it allows opioid administration to be maintained within a narrow therapeutic range, and plasma concentrations can be more precisely controlled than with intravenous or oral administration. Commonly used PCA agents in pediatric patients include morphine, fentanyl, and hydromorphone (Table 5) [18,42]. Nevertheless, PCA dosing regimens should be carefully individualized based on factors such as pain intensity and patient age, and all patients should be closely monitored because of the potential risks of respiratory depression, nausea, vomiting, and sedation [18]. Clinicians should recognize the pharmacological, pharmacokinetic, and pain-monitoring differences related to age and developmental stage in children.

## Conclusion

Acute postoperative pain in pediatric patients is often underestimated and inadequately managed because of developmental differences in pain expression and assessment challenges. The immaturity of the descending inhibitory pathways and increased neuroplasticity can intensify pain perception in children. Effective pain management requires age-appropriate and validated assessment tools that consider cognitive development and situational factors. Given age-related variability in pharmacokinetics and pharmacodynamics, individualized multimodal analgesic strategies with careful dose adjustments should be utilized. They have been shown to improve pain control and recovery outcomes in pediatric patients undergoing surgery.

## Figures and Tables

**Table 1.** Pediatric pain assessment tools

Age group	Assessment method	Commonly used tools	Key assessment items and features
Neonates and infants (0–1 yr)	Physiological and behavioral observation	CRIES	· CRIES: crying, increased oxygen requirement, vital signs (HR/BP), facial expression, sleeplessness
		Neonatal Infant Pain Scale (NIPS)	· NIPS: facial expression, cry, breathing pattern, arms, legs, state of arousal
		Premature Infant Pain Profile (PIPP)	-PIPP: gestational age, behavioral state, HR, and oxygen saturation changes, brow bulge, eye squeeze, nasolabial furrow
Infants and toddlers (1–3 yr)	Behavioral observation	Face, Legs, Activity, Cry, Consolability (FLACC) Scale	· Measures intensity of pain or discomfort
			· Assesses facial expression, leg movement, activity, cry, consolability
Preschool children (3–7 yr)	Behavioral observation+self-report	Objective Pain Scale and Comfort Scale (OPSCS)	· Combines observation and simple self-report
		Facial Pain Scale	- Uses pictorial tools such as facial drawings to help children indicate pain level
		Wong-Baker Faces Pain Rating	
Older children and adolescents (≥7 yr)	Mainly self-report	Visual Analog Scale (VAS)	· Cognitive and linguistic development sufficient for direct pain description
		Numeric Rating Scale (NRS)	· Pain intensity rated on a 0–10 scale
		Other self-report scales	

HR, heart rate; BP, blood pressure.

**Table 2.** Opioids commonly used in pediatric clinical pain management

Drug	Typical intravenous dose (μg/kg)	Usual dosing interval (hr)	Notes
Morphine	Bolus: 50–100	Every 2–4	Nausea, vomiting, sedation, respiratory depression
Fentanyl	Bolus: 0.5–1	Every 2–4	More potent than morphine, sedation, respiratory depression
Hydromorphone	Bolus: 10–15	Every 3–6	Very rapid onset and short duration, chest wall rigidity, sedation, respiratory depression

**Table 3.** Nonopioids commonly used in pediatric clinical pain management

Drug	Typical IV dose	Maximum daily dose	Notes
Acetaminophen	2–12 yr: 12.5 mg/kg every 4 hr or 15 mg/kg every 6 hr (≥15 min infusion)	75 mg/kg/day	Use lower daily limit in liver disease, malnutrition
	≥13 yr, <50 kg: dosing for children aged 2–12 yr	3.75 g/day	
	≥13 yr, ≥50 kg: 650 mg every 4 hr or 1,000 mg every 6 hr	4 g/day	
Ketorolac	2–16 yr: <50 kg: 0.5 mg/kg IV every 6 hr PRN, max 15 mg per dose	60 mg/day	Avoid in renal impairment, bleeding risk, GI ulcer, thrombocytopenia
	≥50 kg: 30 mg IV every 6 hr PRN, max 30 mg per dose		
Ibuprofen	6 mo to <12 yr: 5–10 mg/kg every 6 hr (≥15 min infusion) 12–17 yr: 400 mg IV every 6 hr (≥15 min infusion)	40 mg/kg/day or 2,400 mg/day	Avoid in significant renal dysfunction, active bleeding, platelet dysfunction

IV, intravenous; PRN, pro re nata; GI, gastrointestinal.

**Table 4.** Pediatric peripheral nerve block-local anesthetic dose

Local anesthetic	Concentration (%)	Volume (mL/kg)	Maximum dose (mg/kg)	Expected duration (hr)
Lidocaine	1–2	0.25	3–4	1–2
Bupivacaine	0.2%	0.5	2–2.5	6–12
Ropivacaine	0.2	0.5	2.5	6–12
Levobupivacaine	0.25	0.5	2.5	6–12

**Table 5.** Pediatric patient-controlled analgesia (PCA) protocol with opioids

Drug	PCA bolus dose (μg/kg)	Lockout interval (min)	Basal infusion (μg/kg/hr)
Morphine	10–20	6–10	10-20
Fentanyl	0.2–0.3	5–10	0.3–1
Hydromorphone	2–4	6–10	2–4
